# Response to Allard: A minor (albeit significant) role for voltage-induced calcium release in *Caenorhabditis elegans* muscles

**DOI:** 10.1073/pnas.2415044121

**Published:** 2024-09-16

**Authors:** Luna Gao, Evan Ardiel, Stephen Nurrish, Joshua M. Kaplan

**Affiliations:** ^a^Department of Molecular Biology, Massachusetts General Hospital, Boston, MA 02114; ^b^Department of Neurobiology, Harvard Medical School, Boston, MA 02115; ^c^Program in Neuroscience, Harvard Medical School, Boston, MA 02115

We thank Allard (1) for comments on our paper (2). Voltage-induced calcium release (VICR) is a mechanism whereby voltage-induced conformational changes in type 1 voltage-activated calcium channels (CaV1) (residing in the plasma membrane) are allosterically coupled to activation of calcium channels in the sarcoplasmic reticulum (SR) without requiring external calcium entry (ECE) (3, 4). We used a calcium-activated potassium channel (SLO-2) as an assay to determine whether VICR occurs in *Caenorhabditis elegans* body muscles. We find that SLO-2 activation is mediated by both ECE and VICR, with each mechanism contributing equally. We had two main conclusions. First, VICR occurs in invertebrate muscles, contrary to prior studies suggesting that VICR is restricted to vertebrate skeletal muscles (5). Second, a minor contribution of VICR to calcium transients could be missed in cell types where ECE and calcium-induced calcium release are robust. This is particularly true in studies relying on assays of bulk cytoplasmic calcium levels (e.g., calcium dyes or muscle contraction), including the work from Allard (6). By contrast, SLO-2 activation assays calcium in microdomains surrounding CaV1 channels, which we propose increases sensitivity for detecting VICR. Allard has two criticisms of our conclusions, which we respond to below:

1) *Voltage-dependent SLO-2 activation.* Allard proposes that SLO-2 current observed in the absence of ECE does not reflect VICR-mediated SLO-2 activation but instead results from intrinsic voltage-dependent (but calcium-independent) SLO-2 activation. Allard further notes that voltage-dependent SLO-2 activation could be exaggerated in the absence of external calcium. Three results (all reported in our study) argue against this possibility. First, depolarization induced SLO-2 current was eliminated by the CaV1 antagonist nemadipine. Second, the residual SLO-2 current in the absence of external calcium was eliminated by preventing SR calcium release (using the SERCA inhibitor CPA). Third, blocking ECE with a general CaV antagonist (CdCl_2_) produced a partial loss of SLO-2 current similar to that seen in the absence of external calcium. Thus, voltage-dependent SLO-2 activation cannot account for the residual SLO-2 current observed when ECE is blocked. Finally, Allard’s explanation would require that *egl-19(*ΔVTTL*)*, *shn-1*, *jph-1*, *itr-1,* and *unc-68* mutations do not disrupt VICR-mediated SLO-2 activation (as we propose) but instead alter the intrinsic voltage dependence of SLO-2 channels, which seems very unlikely.
2) *C. elegans VICR (should it exist) is not physiologically significant.* As Allard notes, locomotion was fairly normal in VICR-deficient mutants, suggesting that excitation–contraction coupling was not strongly disrupted. This result suggests that VICR makes a modest contribution to muscle calcium transients. Instead, we propose that VICR-mediated SLO-2 activation (which accounts for roughly 50% of the SLO-2 current) plays an important role in excitation–repolarization coupling. This conclusion is supported by our prior paper showing that muscle action potentials were significantly prolonged in two VICR-deficient mutants, *egl-19(*ΔVTTL*)* and *shn-1* (7), which may not significantly alter locomotion.
